# Availability of ChatGPT to provide medical information for patients with kidney cancer

**DOI:** 10.1038/s41598-024-51531-8

**Published:** 2024-01-17

**Authors:** Joongwon Choi, Jin Wook Kim, Yong Seong Lee, Jong Hyun Tae, Se Young Choi, In Ho Chang, Jung Hoon Kim

**Affiliations:** 1 Department of Urology, Chung-Ang University Gwangmyeong Hospital, Chung-Ang University College of Medicine, Seoul, South Korea; 2 Department of Urology, Chung-Ang University Hospital, Chung-Ang University College of Medicine, Seoul, South Korea; 3 Chung-Ang University Gwangmyeong Hospital, 110 Deokan-Ro, Gwangmyeong-Si, Gyeonggi-Do 14353 South Korea

**Keywords:** Cancer, Computational biology and bioinformatics, Urology

## Abstract

ChatGPT is an advanced natural language processing technology that closely resembles human language. We evaluated whether ChatGPT could help patients understand kidney cancer and replace consultations with urologists. Two urologists developed ten questions commonly asked by patients with kidney cancer. The answers to these questions were produced using ChatGPT. The five-dimension SERVQUAL model was used to assess the service quality of ChatGPT. The survey was distributed to 103 urologists via email, and twenty-four urological oncologists specializing in kidney cancer were included as experts with more than 20 kidney cancer cases in clinic per month. All respondents were physicians. We received 24 responses to the email survey (response rate: 23.3%). The appropriateness rate for all ten answers exceeded 60%. The answer to Q2 received the highest agreement (91.7%, etiology of kidney cancer), whereas the answer to Q8 had the lowest (62.5%, comparison with other cancers). The experts gave low assessment ratings (44.4% vs. 93.3%, p = 0.028) in the SERVQUAL assurance (certainty of total answers) dimension. Positive scores for the overall understandability of ChatGPT answers were assigned by 54.2% of responders, and 70.8% said that ChatGPT could not replace explanations provided by urologists. Our findings affirm that although ChatGPT answers to kidney cancer questions are generally accessible, they should not supplant the counseling of a urologist.

## Introduction

Artificial intelligence (AI) has gained widespread traction across all aspects of our daily lives. The integration of AI with healthcare systems has gradually increased. A significant milestone in this collaboration between physicians and AI was reached on November 30, 2022, with the introduction of OpenAI’s most advanced Chat generative pre-trained transformer (ChatGPT) version. This sophisticated AI chatbot, built on a large language model, is now accessible through a user-friendly web interface that allows free access by the public^1^. As an advanced natural language processing (NLP) technology, it closely emulates human language. This feature is achieved using a deep learning algorithm trained on an extensive dataset containing 175 billion parameters^2^. Although the ChatGPT technology is not new, its notable aspect lies in its availability to the public without additional fees. Moreover, the user-friendly interface and large dataset contribute to the uniqueness of ChatGPT.

Patients with kidney cancer may have concerns about the disease characteristics, diagnostic examinations and available treatment options. While CT scan are commonly used for diagnosing renal cell carcinoma (RCC) with high accuracy (70–80%), patients may question the absence of other diagnostic tests such as biopsies or MRI scans^3^. Additionally, the emergence of new treatment approaches, such as the combination of lenvatinib and pembrolizumab for metastatic RCC, raises questions about the accessibility of state-of-the-art treatments for patients^4^.

The SERVQUAL model is a research tool that assesses how five dimensions—tangibility, reliability, responsiveness, assurance, and empathy—influence customer perception^5^. It is the most popular scale used to describe service quality in hospitals worldwide. It was designed by Zeithamlai, Parasuraman, and Berry in 1985 to evaluate non-medical service quality. The original SERVQUAL scale consisted of 44 questions^6^. The answers to the questions are presented in a five-point Likert scale. SERVQUAL has mainly been used to evaluate the quality of medical services in hospitals and healthcare institutions. It demonstrates patient expectations and contributes to improvements in medical services^7^. It is also suitable for comparing the service quality of different medical facilities^8^. However, SERVQUAL has not yet been used to investigate the reliability of ChatGPT or the effects of ChatGPT responses on patient understanding.

In this study, we evaluated the ability of ChatGPT to understand kidney cancer, including RCC, and to offer suitable recommendations for the general population. By engaging in consultations with urology experts and eliciting responses from ChatGPT through queries, we assessed whether the answers generated by ChatGPT concerning common queries about kidney cancer could augment patients’ comprehension and serve as a substitute for explanations provided by urologists regarding RCC.

## Materials and methods

**Figure 1:**
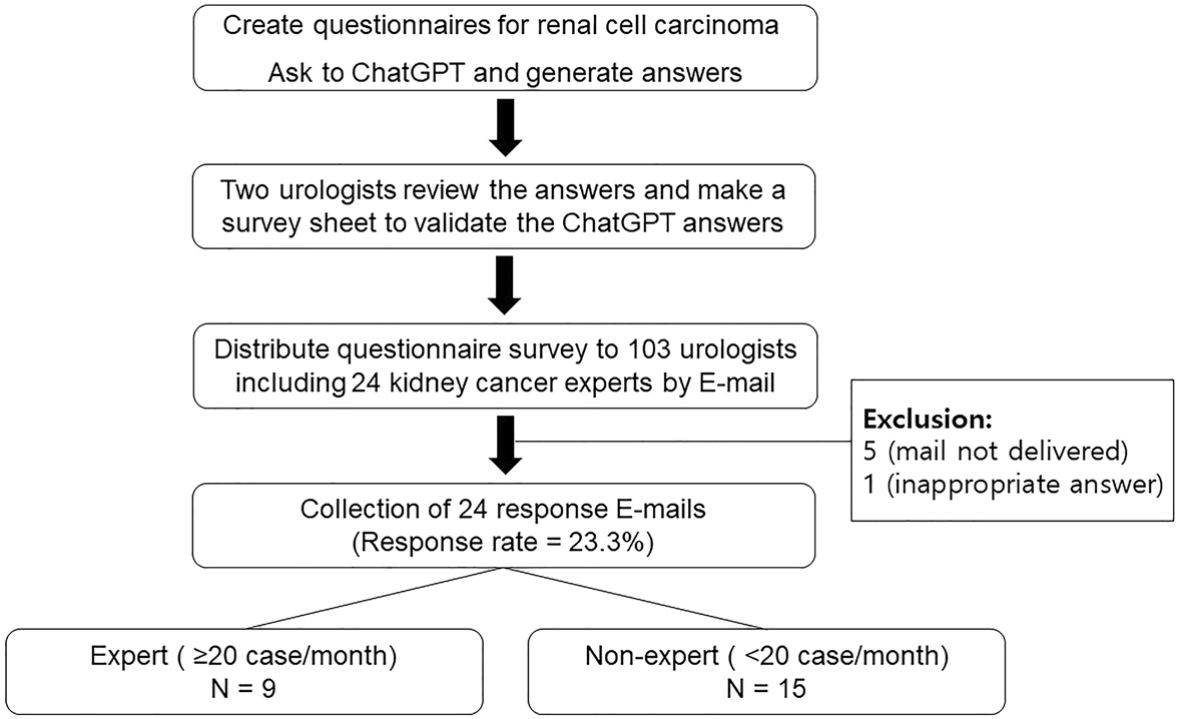
Flow chart of the study. The entire research process involved creating questionnaires and undergoing validation by two urologists. Following validation, E-mail distribution was conducted.

The study utilized ChatGPT, a language model developed by OpenAI in San Francisco, California, USA, based on the GPT-3.5 architecture (last updated in September 2021). A set of ten English-based questions was designed by two urologists from a university hospital. The questions were formulated by referencing the 'People also ask' section when searching for Kidney cancer on Google.com™ and by collaborating to generate questions commonly asked by outpatient individuals, with the aim of avoiding redundancy in overall categories. The questions addressed various aspects of kidney cancer, including symptoms, causes, treatment methods, prevention strategies, genetic effects, incidence rates, treatment of metastatic cancer, differences from other cancers, survival rates, and recurrence rates. The list of questions is presented in Table 1, and the ChatGPT-derived responses are displayed in Fig. 1.

**Table 1:** Question list.

Question number	Asked question	Category
1	What symptoms of kidney cancer can I suspect?	Symptom
2	What causes kidney cancer?	Cause
3	What are some treatments for kidney cancer?	Treatment
4	How to prevent kidney cancer?	Prevention
5	Is kidney cancer genetically affected?	Genetics
6	What is the incidence of kidney cancer?	Incidence
7	I heard that kidney cancer has metastasized, what is the best treatment? Can it be completely cured?	Metastasis
8	What is the difference between kidney cancer and other types of cancer?	Differences
9	What is the survival rate of kidney cancer after treatment?	Survival rate
10	What is the probability that kidney cancer will recur?	Probability

We aimed to measure the quality of ChatGPT answers using simple SERVQUAL questions. SERVQUAL model was conducted covering five dimensions, including tangibility, reliability, response, assessment, and affordability, which allowed respondents to rate ChatGPT answers on a scale of 1–5. We intended to assess levels of online services by adapting the original SERVQUAL model, modifying it to be suitable for evaluating online services. We restructured each category into five items, symbolizing very high/high/normal/low/very low, to facilitate a more fitting evaluation. We attached five SERVQUAL questions to the end of the survey. Moreover, two comprehensive assessment questions were incorporated to assess whether the ChatGPT responses were understandable to patients and whether they could replace the explanations provided by urologists.

The survey, which included the ChatGPT answers and the quality assessment, was distributed via email to 103 urologists, of which 24 were experts. The expert was defined as urologists affiliated with the Korean Urological Oncology Society and the Korean Renal Cancer Research Society (KRoCS) with more than 20 kidney cancer cases in clinic per month. KRoCS members consist of professors of urology specializing in the treatment of kidney cancer at university hospitals who have published research^9–11^. All respondents were physicians and responses were collected using Google Forms (https://docs.google.com/forms).

All statistical analyses were performed using SPSS software (version 27.0; Statistical Package for Social Sciences, Chicago, IL, USA). Student’s t-test was used to compare the means between the expert and non-expert groups, and statistical significance was set at p < 0.05.

The Institutional Review Board of Chung-Ang University Gwangmyeoung Hospital approved this study (approval number: 2310-112-114). Because of its retrospective nature, the need for informed consent was waived by the IRB of the Chung-Ang University Gwangmyeoung Hospital based on the Unites States Department of Health and Human Services code 46.116 for requirements for informed consent. The study was conducted according to the ethical standards recommended by the 1964 Declaration of Helsinki and its later amendments.

## Results

There were 24 responses to the e-mail survey, with a response rate of 23.3%. The demographic characteristics of the respondents are presented in Table 2. Notably, nine experts reported performing over 20 kidney cancer surgeries per month. The answers to all ten questions are provided in Supplementary Table 1.

**Table 2:** Demographic characteristics of the respondents.

Variables	Value
Gender	
Male	24 (100.0%)
Female	0 (0.0%)
Age	
30 s (30–39 years old)	4 (16.7%)
40 s (40–49 years old)	17 (70.8%)
50 s (50–59 years old)	3 (12.5%)
Types of medical institutions	
Clinic	7 (29.2%)
Public health center	1 (4.2%)
Secondary hospital	1 (4.2%)
University or tertiary hospital	15 (62.5%)
Location of medical institute	
Seoul	7 (29.2%)
Gyeonggi Province	6 (25.0%)
Chungcheong Province	3 (12.5%)
Gyeongsang Province	3 (12.5%)
Jeolla Province	4 (16.7%)
Jeju Province	1 ( 4.2%)
Average kidney cancer patients per months	
10 to 20 people	3 (12.5%)
20 to 40 people	1 ( 4.2%)
Less than 10 people	12 (50.0%)
More than 40 people	8 (33.3%)

**Figure 2:**
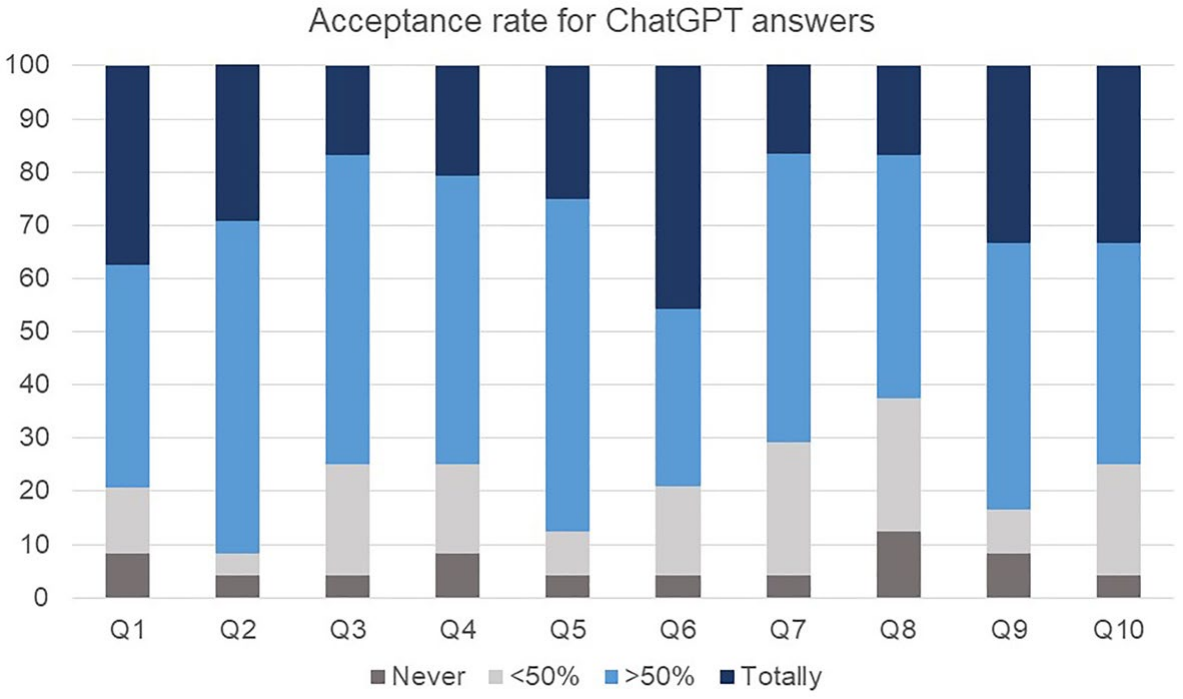
Acceptance rate for ChatGPT answers. The evaluation of ChatGPT's responses by urologists indicates that the positive evaluation rate (combined dark blue + light blue) exceeded 60% across all responses.

The overall positive evaluation rate of the urologists for all ten answers was 77.9%, ranging from 62.5 to 91.7%, as illustrated in Fig. 2. The answer to question 2, which asked about the causes of kidney cancer, received the highest positive evaluation rate of 91.7%, whereas answer 8, pertaining to the differences between kidney cancer and other types of cancer, received the lowest positive evaluation rate of 62.5%. Notably, eight of the 10 answers achieved a positive evaluation rate of ≥ 75%.

Applying the SERVQUAL model (Table 3), the assessment of reliability (average score of 3.4/5.0, p = 0.004) and responsiveness (average score of 3.2/5.0, p < 0.001) yielded lower points compared to tangibility (average score of 4.1/5.0). There was no statistical difference in the positive evaluation rate between expert and non-expert respondents for most of the survey. However, for the assurance dimension, which evaluated the certainty of the total answers provided, the experts gave the lowest positive evaluation rate (44.4% vs. 93.3%, p = 0.28; Table 4).

**Table 3:** Service quality assessment; SERVQUAL model.

Multi-dimension of ChatGPT service quality	Value	Point	Average	p-value*
Tangibility; Structural solidity of total answers				
It is well structured for logical construction	4 (17.4%)	5	4.1	-
It is structured relatively logically	17 (73.9%)	4		
It's normal	2 (8.7%)	3		
Reliability; Reliability of total answers				
The answer is accurate and reliable	1 (4.2%)	5	3.4	0.004
It is generally reliable	13 (54.2%)	4		
It's normal	5 (20.8%)	3		
There is a part that is not being able to be trusted	4 (16.7%)	2		
The accuracy or reliability of the answer is poor	1 (4.2%)	1		
Responsiveness; Latest knowledge reflectivity of total answers				
It reflects the latest guidelines	2 (8.3%)	5	3.2	< 0.001
Contain the recent knowledge	6 (25.0%)	4		
It's normal	13 (54.2%)	3		
It only reflects past knowledge	1 (4.2%)	2		
Lack the latest knowledge	2 (8.3%)	1		
Assurance; Certainty of total answers				
It is stable enough that ordinary people can trust and use it	2 (8.3%)	5	3.7	0.076
Be useful for reference	16 (66.7%)	4		
It's normal	4 (16.7%)	3		
It should not be trusted and used by the public	2 (8.3%)	1		
Empathy; Empathy of total answers				
It is an appropriate answer that accurately understands the meaning of the question and considers the user	4 (16.7%)	5	3.9	0.262
It has some understanding of the user who asked	13 (54.2%)	4		
It's normal	7 (29.2%)	3		

*Compared with Tangibility parameter.

**Table 4:** Response differences according to expert (≥ 20 case/month) or non-expert (< 20 case/month).

	Expert	Non-expert	p-value
	(N = 9)	(N = 15)	
Q1. Symptoms			0.497
I totally agree with the answer	3 (33.3%)	6 (40.0%)	
Overall (> 50%) I agree with the answer	4 (44.4%)	6 (40.0%)	
The answer is insufficient and insufficient (< 50%)	2 (22.2%)	1 (6.7%)	
There is an error in the answer and should not be used for actual medical treatment	0 (0.0%)	2 (13.3%)	
Q2. Causes			0.467
I totally agree with the answer	2 (22.2%)	5 (33.3%)	
Overall (> 50%) I agree with the answer	6 (66.7%)	9 (60.0%)	
The answer is insufficient and insufficient (< 50%)	1 (11.1%)	0 ( 0.0%)	
There is an error in the answer and should not be used for actual medical treatment	0 ( 0.0%)	1 ( 6.7%)	
Q3. Treatment			0.789
I totally agree with the answer	1 (11.1%)	3 (20.0%)	
Overall (> 50%) I agree with the answer	6 (66.7%)	8 (53.3%)	
The answer is insufficient and insufficient (< 50%)	2 (22.2%)	3 (20.0%)	
There is an error in the answer and should not be used for actual medical treatment	0 (0.0%)	1 (6.7%)	
Q4. Prevention			0.329
I totally agree with the answer	1 (11.1%)	4 (26.7%)	
Overall (> 50%) I agree with the answer	4 (44.4%)	9 (60.0%)	
The answer is insufficient and insufficient (< 50%)	3 (33.3%)	1 ( 6.7%)	
There is an error in the answer and should not be used for actual medical treatment	1 (11.1%)	1 ( 6.7%)	
Q5. Genetics			0.524
I totally agree with the answer	3 (33.3%)	3 (20.0%)	
Overall (> 50%) I agree with the answer	6 (66.7%)	9 (60.0%)	
The answer is insufficient and insufficient (< 50%)	0 (0.0%)	2 (13.3%)	
There is an error in the answer and should not be used for actual medical treatment	0 (0.0%)	1 (6.7%)	
Q6. Incidence			0.704
I totally agree with the answer	4 (44.4%)	7 (46.7%)	
Overall (> 50%) I agree with the answer	4 (44.4%)	4 (26.7%)	
The answer is insufficient and insufficient (< 50%)	1 (11.1%)	3 (20.0%)	
There is an error in the answer and should not be used for actual medical treatment	0 (0.0%)	1 (6.7%)	
Q7. Metastasis treatment			0.282
I totally agree with the answer	0 (0.0%)	4 (26.7%)	
Overall (> 50%) I agree with the answer	6 (66.7%)	7 (46.7%)	
The answer is insufficient and insufficient (< 50%)	3 (33.3%)	3 (20.0%)	
There is an error in the answer and should not be used for actual medical treatment	0 (0.0%)	1 (6.7%)	
Q8. Compare with other cancer			0.430
I totally agree with the answer	1 (11.1%)	3 (20.0%)	
Overall (> 50%) I agree with the answer	5 (55.6%)	6 (40.0%)	
The answer is insufficient and insufficient (< 50%)	3 (33.3%)	3 (20.0%)	
There is an error in the answer and should not be used for actual medical treatment	0 (0.0%)	3 (20.0%)	
Q9. Survival rate			0.700
I totally agree with the answer	3 (33.3%)	5 (33.3%)	
Overall (> 50%) I agree with the answer	5 (55.6%)	7 (46.7%)	
The answer is insufficient and insufficient (< 50%)	1 (11.1%)	1 (6.7%)	
There is an error in the answer and should not be used for actual medical treatment	0 (0.0%)	2 (13.3%)	
Q10. Recur			0.524
I totally agree with the answer	2 (22.2%)	6 (40.0%)	
Overall (> 50%) I agree with the answer	4 (44.4%)	6 (40.0%)	
The answer is insufficient and insufficient (< 50%)	3 (33.3%)	2 (13.3%)	
There is an error in the answer and should not be used for actual medical treatment	0 (0.0%)	1 (6.7%)	
Tangibility; Structural solidity of total answers			0.210
It is structured relatively logically	8 (88.9%)	9 (64.3%)	
It is well structured for logical construction	0 (0.0%)	4 (28.6%)	
It's normal	1 (11.1%)	1 (7.1%)	
Reliability; Reliability of total answers			0.234
It is generally reliable	4 (44.4%)	9 (60.0%)	
It's normal	4 (44.4%)	1 (6.7%)	
The accuracy or reliability of the answer is poor	0 (0.0%)	1 (6.7%)	
The answer is accurate and reliable	0 (0.0%)	1 (6.7%)	
There is a part that is not being able to be trusted	1 (11.1%)	3 (20.0%)	
Responsiveness; Latest knowledge reflectivity of total answers			0.664
Contain the latest knowledge	1 (11.1%)	5 (33.3%)	
It only reflects past knowledge	0 (0.0%)	1 (6.7%)	
It reflects the latest guidelines	1 (11.1%)	1 (6.7%)	
It's normal	6 (66.7%)	7 (46.7%)	
Lack the latest knowledge	1 (11.1%)	1 (6.7%)	
Assurance; Certainty of total answers			0.028
Be useful for reference	4 (44.4%)	12 (80.0%)	
It is stable enough that ordinary people can trust and use it	0 (0.0%)	2 (13.3%)	
It should not be trusted and used by the public	1 (11.1%)	1 (6.7%)	
It's normal	4 (44.4%)	0 (0.0%)	
Empathy; Empathy of total answers			0.127
I have some understanding of the user who asked	7 (77.8%)	6 (40.0%)	
It is an appropriate answer that accurately understands the meaning of the question and considers the user	0 (0.0%)	4 (26.7%)	
It's normal	2 (22.2%)	5 (33.3%)	
Comprehensive evaluation 1; comfortable to understand			0.594
It's hard to understand	0 (0.0%)	1 (6.7%)	
It's normal	4 (44.4%)	4 (26.7%)	
It's relatively easy to understand	4 (44.4%)	5 (33.3%)	
It's very easy to understand	1 (11.1%)	3 (20.0%)	
It's very hard to understand	0 ( 0.0%)	2 (13.3%)	
Comprehensive evaluation 2; Could replace the urologist’s explanation			0.297
No, it cannot replace the urologist's explanation	8 (88.9%)	9 (60.0%)	
Yes, I think it can be replaced	1 (11.1%)	6 (40.0%)	

**Figure 3:**
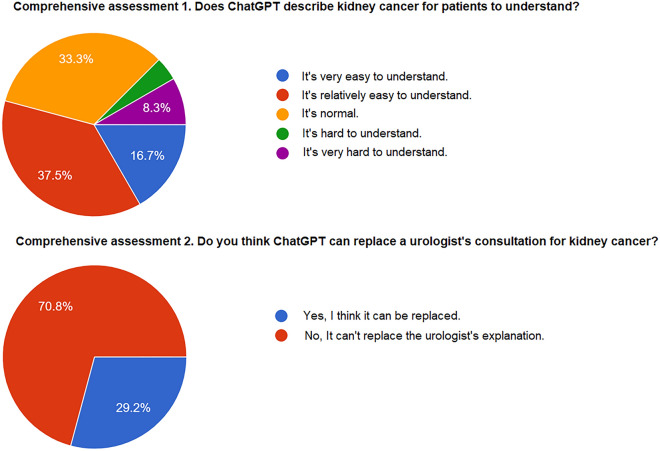
Comprehensive assessment for ChatGPT answers. In the comprehensive assessment of ChatGPT's responses, the positive evaluation rate for being understandable reached 87.5%, but it was noted that it couldn't fully replace the consultation by urologists, with a response rate of 70.8%.

In the comprehensive assessment, 54.2% of the respondents expressed a positive evaluation (indicating that responses were better than normal) regarding ChatGPT’s ability to provide comprehensible responses (Fig. 3). However, only 29.2% of the urologists believed that ChatGPT-derived responses could replace explanations provided by urologists.

## Discussion

Our survey of urologists evaluating ChatGPT responses to questions about kidney cancer resulted in an overall positive rating of 77.9%, with an excellent positive evaluation rating of 91.7% for answers to questions such as “causes of kidney cancer,” for which an internet search is possible. However, it received relatively low scores for reliability (3.4/5.0) and responsiveness to the latest insights (3.2/5.0). This reflects the fact that ChatGPT only incorporates knowledge as of September 2021 and cannot include the latest treatment trends. Further, 30% acceptability-as-a-surrogate rate in 80% positive evaluations is a significant difference. Perhaps it could be attributed to an issue regarding the doctor's privilege or rights and follow-up research is necessary. Additionally, when examining the responses of expert and non-expert physicians with a monthly caseload of 20 or more kidney cancer cases, there were no significant differences observed except in the Assurance (certainty of total answers) category. This suggests that the expert group may have responded more sensitively to the characteristics of ChatGPT, which only reflects information up to 2021.

ChatGPT is a convenient and powerful tool for providing medical information. ChatGPT could potentially serve as a tool to provide clinical guidance to patients, suggest treatment options based on guidelines, and be utilized for medical education^12–14^. A few studies have used ChatGPT for the treatment or assessment of urological diseases. Coskun et al. assessed the quality of ChatGPT information on prostate cancer and demonstrated that ChatGPT information was lacking in terms of accuracy and interpretation^15^. To improve a patient’s deep understanding, the authors suggested the need for improved reliability, evidenced-based information, understanding of patient emotions or experiences, and brevity. Davis et al. examined the appropriateness of NLP for urological diseases and reported that there are limitations to the medical information on NLP^16^. Urologists pointed out that vital information was missing from the content provided by ChatGPT. Despite these results, the use of ChatGPT is gradually expanding in the real world.

Doubts regarding reliability likely stem from ChatGPT operating as a generative model that provides appropriate responses in interactive situations. It utilizes natural language processing techniques to understand user input and generate responses based on the training received from a large-scale text dataset. Although the GPT strives to learn patterns, context, and meaning to produce natural conversations, it does not always provide accurate or perfect answers because it relies on pre-trained data. In other words, ChatGPT does not generate “true knowledge-based answers” and does not take responsibility for the responses.

The use of AI or machine learning in urology is common. As ChatGPT is not an AI application trained using a specialized medical database, it may be inaccurate or misleading in answering medical questions^17^. Howard et al. assessed infection consultations and the selection of antimicrobial agents and concluded that answers from ChatGPT were inadequate and inconsistent and recommended a qualitative modification that can be applied to medical specialties^18^. Zhou et al. assessed the appropriateness of ChatGPT in urology and reported that ChatGPT was generally consistent and well-aligned with the guidelines for urological diseases^19^. Davis et al. investigated the appropriateness and readability of ChatGPT responses to urology-related medical inquiries^16^. The authors used 18 urological questions based on Google Trends, covering the categories of malignancy, emergency, and benign diseases. They suggested that vital information lacking in the ChatGPT answers was a limitation.

Among the five dimensions of SERVQUAL questions, only assurance demonstrated a significant difference between the experts and general urologists (p = 0.028). Most general urologists responded that the ChatGPT answers were reliable and convincing (93.3%); however, approximately 55.5% of the experts on kidney cancer thought the answers were unreliable. The difference in the responses on assurance between the two groups likely stems from the knowledge of the kidney cancer expert group. Although ChatGPT has sufficient function to deliver information about kidney cancer to patients, we suggest that it lacks specialized medical knowledge.

This study had several limitations. First, the low response rate (23.3%) and relatively small sample size are notable limitations. Additionally, it is worth mentioning that only approximately 50% of the respondents were experts who performed more than 20 kidney cancer surgeries per month. Therefore, it can be considered a drawback that the sample may not fully represent all urologists. Further research involving a larger group of expert respondents is required to address these limitations. Second, the overall responses from ChatGPT tended to repetitively explain general information when answering the questions. Evaluating this aspect using the existing SERVQUAL model (tangibility, reliability, responsiveness, assurance, and empathy) may be inappropriate. Therefore, evaluation metrics that specifically assess response specificity are required. Third, this study did not include Bard (Google), Claude 2 or Llama 2, another NLP technology model with a public face; Therefore, it is unclear whether the responses obtained reflect the general characteristics of all NLP technology models. Fourth, our survey only involves questions directed at physicians, excluding input from patients. In future research, targeting patients could provide results that better reflect real-world practice. Lastly, GPT-4 was promptly launched on March 14, 2023, subsequent to GPT-3.5, and is regarded as a more advanced model in terms of its performance and capabilities for ChatGPT. Although we recognize the widespread availability and enhanced accessibility of GPT-3.5 due to its free usage, we acknowledge that it may have limitations in delivering comprehensive information compared to the more advanced GPT-4. We did not conduct a specific inquiry into the accuracy of the information provided by ChatGPT through an examination of the source text.

The application of ChatGPT in the medical or healthcare environment is currently in its nascent stages. Our findings shed light on the potential of AI-driven language models such as ChatGPT to assist in medical information dissemination while emphasizing the importance of maintaining the role of expert human healthcare providers in patient care and education.

## Conclusions

According to the urologists surveyed, the ChatGPT answers to common questions regarding kidney cancer were widely understandable and accessible. However, most participants, particularly the group of experts who exhibited a lower level of consensus in the dimension of assurance, concluded that ChatGPT could not entirely substitute for the guidance of a urologist.

### Supplementary Information

Supplementary Table 1.

## Data Availability

The datasets generated during and/or analysed during the current study are available from the corresponding author on reasonable request.
