# Supplementary material for: Availability of ChatGPT to provide medical information for patients with kidney cancer
Source: Sci Rep. 2024 Jan 17;14:1542. doi: 10.1038/s41598-024-51531-8 (PMC10794224; doi:10.1038/s41598-024-51531-8)
Supplement: Supplementary file 1 — Supplementary Table 1. [file 41598_2024_51531_MOESM1_ESM.docx]

Supplementary Table 1. ChatGPT Total Q & A

| **Q1. What symptoms of kidney cancer can I suspect?** |
| --- |
| **A1.** Kidney cancer, also known as renal cell carcinoma, may not cause any noticeable symptoms in its early stages. As the cancer grows, symptoms may begin to appear. Some of the common symptoms of kidney cancer include: Blood in urine / Abdominal pain or lower back pain / Unexplained weight loss / Fatigue / Loss of appetite / Swelling in the legs or ankles / Anemia (low red blood cell count) / High blood pressure It is important to note that these symptoms may be caused by other medical conditions as well, and the presence of one or more of these symptoms does not necessarily mean that you have kidney cancer. If you experience any of these symptoms, it is important to consult with a healthcare professional for a proper diagnosis. |
| **Q2. What causes kidney cancer?** |
| **A2.** The exact cause of kidney cancer is not fully understood, but there are several factors that can increase the risk of developing this disease. Some of the common risk factors for kidney cancer include: Smoking: Smoking is one of the most significant risk factors for kidney cancer. People who smoke have a higher risk of developing this disease compared to non-smokers. Age: The risk of kidney cancer increases with age, and it is more common in people over the age of 60. Obesity: People who are overweight or obese have a higher risk of developing kidney cancer. High blood pressure: High blood pressure is a risk factor for kidney cancer. Family history: If a close family member has had kidney cancer, the risk of developing the disease may be higher. Occupational exposure: Certain occupational exposures, such as to chemicals like cadmium, may increase the risk of kidney cancer. Kidney disease: People with certain kidney diseases, such as polycystic kidney disease, have an increased risk of developing kidney cancer.  It is important to note that not everyone with these risk factors will develop kidney cancer, and some people without these risk factors may still develop the disease. Regular medical checkups and early detection can improve the chances of successful treatment for kidney cancer. |
| **Q3. What are some treatments for kidney cancer?** |
| **A3.** The treatment for kidney cancer depends on several factors, such as the stage and location of the cancer, the patient's overall health, and the presence of any other medical conditions. Some common treatments for kidney cancer include: Surgery: Surgery is the most common treatment for kidney cancer. The surgeon may remove part or all of the affected kidney, along with the tumor and surrounding tissue. Radiation therapy: Radiation therapy uses high-energy X-rays or other forms of radiation to kill cancer cells. It may be used to treat kidney cancer that has spread to other parts of the body. Targeted therapy: Targeted therapy is a type of treatment that uses drugs to target specific molecules that help cancer cells grow and divide. These drugs may be used to treat advanced kidney cancer that has spread to other parts of the body. Immunotherapy: Immunotherapy is a type of treatment that uses drugs to stimulate the body's immune system to fight cancer. This treatment may be used to treat advanced kidney cancer. Chemotherapy: Chemotherapy is a type of treatment that uses drugs to kill cancer cells. It is not typically used to treat kidney cancer, but may be used in rare cases.  It is important to note that the treatment plan for kidney cancer should be tailored to each individual patient's needs, and should be determined in consultation with their healthcare team. |
| **Q4. How to prevent kidney cancer?** |
| **A4.** While there is no surefire way to prevent kidney cancer, there are some steps you can take to reduce your risk: Quit smoking: Smoking is a significant risk factor for kidney cancer, and quitting smoking can lower your risk. Maintain a healthy weight: Being overweight or obese increases your risk of kidney cancer. Adopting a healthy lifestyle that includes regular exercise and a balanced diet can help maintain a healthy weight. Control high blood pressure: High blood pressure can increase your risk of kidney cancer. To control high blood pressure, you can adopt a healthy diet, exercise regularly, and take medication as prescribed by your doctor. Manage diabetes: People with diabetes are at a higher risk of developing kidney cancer. Managing your blood sugar levels through diet, exercise, and medication can help reduce this risk. Limit exposure to certain chemicals: Exposure to certain chemicals, such as asbestos, can increase your risk of kidney cancer. If you work with chemicals, be sure to follow proper safety protocols. Get screened: If you have a family history of kidney cancer or other risk factors, talk to your doctor about screening tests. Early detection and treatment can significantly improve your chances of recovery. |
| **Q5. Is kidney cancer genetically affected?** |
| **A5.** There is evidence to suggest that genetics can play a role in the development of kidney cancer. In some cases, kidney cancer can be caused by inherited genetic mutations or changes in certain genes that increase the risk of developing the disease. There are several genetic syndromes that are associated with an increased risk of kidney cancer, including: Von Hippel-Lindau (VHL) syndrome: People with VHL syndrome have a genetic mutation that increases their risk of developing kidney cancer, as well as other types of tumors. Hereditary papillary renal cell carcinoma (HPRC): HPRC is caused by mutations in the MET gene and can increase the risk of developing kidney cancer. Birt-Hogg-Dubé (BHD) syndrome: BHD syndrome is caused by mutations in the FLCN gene and can increase the risk of developing kidney cancer, as well as other types of tumors. Hereditary leiomyomatosis and renal cell cancer (HLRCC): HLRCC is caused by mutations in the FH gene and can increase the risk of developing kidney cancer.  If you have a family history of kidney cancer or any of these genetic syndromes, you may be at increased risk of developing the disease. It is important to discuss your family history with your healthcare provider and consider genetic counseling to determine if you should undergo genetic testing. |
| **Q6. What is the incidence of kidney cancer?** |
| **A6.** The incidence of kidney cancer, also known as renal cell carcinoma, varies by country and region. According to the World Cancer Research Fund, kidney cancer is the 14th most commonly diagnosed cancer in the world, accounting for 2% of all new cancer cases. In the United States, kidney cancer is the 7th most common cancer among men and the 10th most common cancer among women. The American Cancer Society estimates that there will be approximately 76,080 new cases of kidney cancer and 13,780 deaths from kidney cancer in the United States in 2022. The incidence of kidney cancer has been increasing over the past few decades, possibly due to improvements in imaging technology leading to earlier detection, as well as changes in lifestyle factors such as smoking and obesity. It is important to note that while kidney cancer is relatively rare compared to other cancers, it can still have serious health consequences if not diagnosed and treated promptly. Risk factors for kidney cancer include smoking, obesity, high blood pressure, and a family history of kidney cancer. |
| **Q7. I heard that kidney cancer has metastasized, what is the best treatment? Can it be completely cured?** |
| **A7.** When kidney cancer has metastasized, meaning it has spread beyond the kidney to other parts of the body, it is typically classified as advanced or stage IV kidney cancer. While advanced kidney cancer cannot be completely cured, treatment options are available to help manage the cancer and improve quality of life. The treatment for metastatic kidney cancer may involve a combination of surgery, targeted therapy, immunotherapy, radiation therapy, and/or chemotherapy. The goal of treatment is to slow the progression of the cancer, reduce symptoms, and improve quality of life. In some cases, targeted therapy and immunotherapy can be very effective in controlling the growth and spread of metastatic kidney cancer, and can help extend a patient's life. Some patients may also benefit from surgery to remove metastases in specific locations, such as the lungs or bones. It is important to note that the best treatment for metastatic kidney cancer varies depending on each individual's specific case and response to treatment. The healthcare team will work closely with the patient to determine the most appropriate treatment plan based on their medical history, overall health, and preferences. While advanced kidney cancer cannot be completely cured, treatment can help manage the cancer and improve quality of life for many patients. It is important for patients with metastatic kidney cancer to discuss their treatment options and prognosis with their healthcare team. |
| **Q8. What is the difference between kidney cancer and other types of cancer?** |
| **A8.** Kidney cancer, also known as renal cell carcinoma, is a type of cancer that originates in the cells of the kidneys. Like other types of cancer, it is characterized by the uncontrolled growth and spread of abnormal cells. One key difference between kidney cancer and other types of cancer is the location where the cancer originates. Kidney cancer originates in the cells of the kidneys, while other types of cancer can originate in different parts of the body such as the lungs, breast, colon, or prostate. Another difference is the type of cells involved. Kidney cancer most commonly arises from cells in the lining of small tubes in the kidney called renal tubules. Other types of cancer can arise from a variety of cell types, including glandular cells, squamous cells, and melanocytes. Symptoms of kidney cancer can include blood in the urine, flank pain, weight loss, and fatigue. Treatment options for kidney cancer depend on the stage and extent of the disease and may include surgery, radiation therapy, chemotherapy, targeted therapy, or immunotherapy. Overall, while kidney cancer shares some similarities with other types of cancer in terms of its effects on the body, its origin and cell type make it unique. It is important to receive an accurate diagnosis and appropriate treatment for any type of cancer. |
| **Q9. What is the survival rate of kidney cancer after treatment?** |
| **A9.** The survival rate for kidney cancer after treatment depends on several factors, such as the stage and type of cancer, the patient's overall health, and the type of treatment received. Here are some general statistics on the five-year survival rate for kidney cancer based on the stage of the disease: Stage I: About 81% of people with stage I kidney cancer survive for at least five years after diagnosis. Stage II: About 74% of people with stage II kidney cancer survive for at least five years after diagnosis. Stage III: About 53% of people with stage III kidney cancer survive for at least five years after diagnosis. Stage IV: About 8% of people with stage IV kidney cancer survive for at least five years after diagnosis. It's important to note that survival rates are just general statistics and can vary depending on individual factors. Additionally, advances in medical treatment and technology may improve survival rates over time. It's important to talk to your doctor about your specific diagnosis and prognosis. |
| **Q10. What is the probability that kidney cancer will recur?** |
| **A10.** The probability that kidney cancer will recur, or come back, varies depending on several factors such as the stage of the cancer at diagnosis, the size of the tumor, and whether the cancer has spread beyond the kidney. The risk of recurrence is generally higher for patients with more advanced or aggressive forms of kidney cancer. According to the American Cancer Society, the five-year relative survival rate for kidney cancer is approximately 75% for all stages combined. However, this statistic is based on data from patients diagnosed several years ago and may not reflect the most recent advances in treatment.  For patients who have undergone surgery for kidney cancer, the risk of recurrence is highest in the first two years following treatment. The risk of recurrence decreases over time, but patients should still be monitored closely for several years after treatment.  It is important for patients who have been treated for kidney cancer to undergo regular follow-up appointments with their healthcare team to monitor for any signs of recurrence. If the cancer does recur, further treatment options may be available, such as surgery, radiation therapy, targeted therapy, or immunotherapy.  Overall, the risk of recurrence for kidney cancer varies depending on individual factors and requires close monitoring and management by a healthcare team. |
